# Nationwide Distribution of Bovine Influenza D Virus Infection in Japan

**DOI:** 10.1371/journal.pone.0163828

**Published:** 2016-09-28

**Authors:** Taisuke Horimoto, Takahiro Hiono, Hirohisa Mekata, Tomoha Odagiri, Zhihao Lei, Tomoya Kobayashi, Junzo Norimine, Yasuo Inoshima, Hirokazu Hikono, Kenji Murakami, Reiichiro Sato, Hironobu Murakami, Masahiro Sakaguchi, Kazunori Ishii, Takaaki Ando, Kounosuke Otomaru, Makoto Ozawa, Yoshihiro Sakoda, Shin Murakami

**Affiliations:** 1 Department of Veterinary Microbiology, Graduate School of Agricultural and Life Sciences, The University of Tokyo, 1-1-1 Yayoi, Bunkyo-ku, Tokyo, 113–8657, Japan; 2 Laboratory of Microbiology, Graduate School of Veterinary Medicine, Hokkaido University, Kita 18 Nishi 9, Kita-ku, Sapporo, 060–0818, Japan; 3 Organization for Promotion of Tenure Track, University of Miyazaki, 1–1 Gakuen Kibanadai-nishi, Miyazaki, 889–2192, Japan; 4 Center for Animal Disease Control, University of Miyazaki, 1–1 Gakuen Kibanadai-nishi, Miyazaki, 889–2192, Japan; 5 Laboratory of Food and Environmental Hygiene, United Graduate School of Veterinary Sciences, Gifu University, 1–1 Yanagido, Gifu, 501–1193, Japan; 6 Laboratory of Veterinary Microbiology, Co-department of Veterinary Medicine, Iwate University, 3-18-8 Ueda, Morioka, Iwate, 020–8550, Japan; 7 Laboratory of Internal Medicine 3, School of Veterinary Medicine, Azabu University, 1-17-71 Fuchinobe, Chuo-ku, Sagamihara, Kanagawa, 252–5201, Japan; 8 Laboratory of Animal Health 2, School of Veterinary Medicine, Azabu University, 1-17-71 Fuchinobe, Chuo-ku, Sagamihara, Kanagawa, 252–5201, Japan; 9 Laboratory of Microbiology 1, School of Veterinary Medicine, Azabu University, 1-17-71 Fuchinobe, Chuo-ku, Sagamihara, Kanagawa, 252–5201, Japan; 10 Ishii Veterinary Clinic, 4-4-25 Nakamikunigaoka, Sakai-ku, Sakai, 590–0022, Osaka, Japan; 11 Laboratory of Theriogenology, Joint Faculty of Veterinary Medicine, Kagoshima University, 1-21-24 Korimoto, Kagoshima, 890–0065, Japan; 12 United Graduate School of Veterinary Science, Yamaguchi University, 1677–1 Yoshida, Yamaguchi, 753–8515, Japan; 13 Veterinary Teaching Hospital, Joint Faculty of Veterinary Medicine, Kagoshima University, 1-21-24 Korimoto, Kagoshima, 890–0065, Japan; 14 Laboratory of Animal Hygiene, Joint Faculty of Veterinary Medicine, Kagoshima University, 1-21-24 Korimoto, Kagoshima, 890–0065, Japan; 15 Transboundary Animal Diseases Control and Research Center, Joint Faculty of Veterinary Medicine, Kagoshima University, 1-21-24 Korimoto, Kagoshima, 890–0065, Japan; Memorial University of Newfoundland, CANADA

## Abstract

Cattle are major reservoirs of the provisionally named influenza D virus, which is potentially involved in the bovine respiratory disease complex. Here, we conducted a serological survey for the influenza D virus in Japan, using archived bovine serum samples collected during 2010–2016 from several herds of apparently healthy cattle in various regions of the country. We found sero-positive cattle across all years and in all the prefectural regions tested, with a total positivity rate of 30.5%, although the positivity rates varied among regions (13.5–50.0%). There was no significant difference in positivity rates for Holstein and Japanese Black cattle. Positivity rates tended to increase with cattle age. The herds were clearly divided into two groups: those with a high positive rate and those with a low (or no) positive rate, indicating that horizontal transmission of the virus occurs readily within a herd. These data demonstrate that bovine influenza D viruses have been in circulation for at least 5 years countrywide, emphasizing its ubiquitous distribution in the cattle population of Japan.

## Introduction

The provisionally named influenza D virus was first isolated as an influenza C-like virus from pigs with respiratory illness at Oklahoma, USA, in 2011 [1, 2]. However, epidemiological analyses indicated that cattle are the major reservoirs of this virus [2, 3], and that it is potentially involved in the bovine respiratory disease complex [4]. The high morbidity and mortality related to this disease in feedlot cattle are caused by multiple factors including co-infection with several viruses and bacteria. Influenza D viruses were also detected in cattle and pigs with respiratory disease (and in some healthy cattle) in China [5], France [6], Italy [7], and Mexico [4], suggesting their global geographic distribution. More recently, we reported that a herd of cattle in Japan encountered an infection caused by influenza D virus, as revealed by serological evidence using paired serum samples collected before and after the onset of respiratory symptoms and by detection of the virus genome in nasal swab samples from the diseased animals [8]. In addition, the influenza D virus appears to infect both sheep and goats [9], and experimentally infected guinea pigs [10]. However, its zoonotic potential is still undefined owing to limited relevant research [1, 11], even though this virus, like the human influenza C virus, is known to use 9-*O*-acetylated sialic acid as its cellular receptor [2, 12].

In this study, to confirm the circulation of bovine influenza D virus in Japan, we conducted a serological survey, using archived serum samples collected from several herds of cattle in various regions of the country.

## Materials and Methods

### Cells and viruses

Swine testicle (ST) cells (ATCC® CRL-1746) were maintained at 37°C in Dulbecco’s modified Eagle’s medium (DMEM) with 10% fetal calf serum (FCS). Bovine kidney (MDBK) cells were maintained at 37°C in DMEM with 5% FCS. The influenza D reference strain D/swine/OK/1334/2011 (D/OK) [1] was kindly provided by Dr. Benjamin Hause (Kansas State University). The virus was propagated in ST or MDBK cells cultured in serum-free medium in the presence of TPCK-trypsin (1 μg/mL).

### Samples

**Fig 1:**
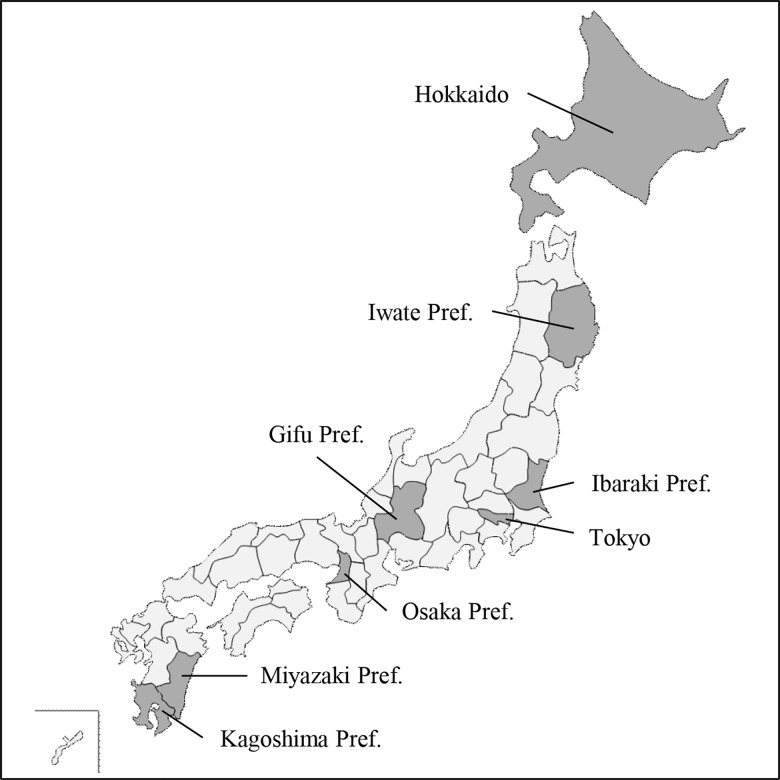
Geographical location of the prefectures in Japan covering the region of cattle sample collection. The location of Ibaraki Prefecture where we first detected the influenza D infection [8] is also shown. This figure is similar but not identical to the original image (www.freemap.jp), and is therefore for illustrative purposes only.

Bovine serum or plasma samples were obtained from several herds in Hokkaido, Iwate, Tokyo, Gifu, Osaka, Miyazaki, and Kagoshima Prefectures covering nearly the entire geographical region of Japan (Fig 1). Samples from herds in Hokkaido, Tokyo, Gifu, and Osaka were collected from Holstein cattle and those from herds in Iwate, Miyazaki, and Kagoshima were collected from Japanese Black beef cattle (Table 1). These samples were originally collected from apparently healthy animals at the time of collection, for research or official purposes involving routine tests for potential diseases such as foot-and-mouth disease, bovine leukemia, and mastitis, and had been stored at -20°C. Although brief records on sex, birth date, and inland movement for each animal in some herds are available, no records showing the clinical history were maintained for most animals. The Committee of Animal Experiments at Graduate School of Agricultural and Life Sciences, The University of Tokyo, permitted our work (Permit Number: P12-652).

**Table 1:** Summary of the serological survey for influenza D virus infection in Japanese cattle.

Prefecture	Cattle	Year	Number of farms	Number of samples	Number of positive farms	Number of positive samples	Positivity rate (%)	Positivity rate in Prefecture (%)
Hokkaido	Holstein	2012	≥8	20	≥4	8	40.0	28.1
		2013	≥104	174	≥45	46	26.4	
		2014	5	5	2	2	40.0	
Iwate	Japanese Black	2015	1	50	1	10	20.0	20.0
Tokyo	Holstein	2014	1	66	1	30	45.5	45.5
Gifu	Holstein	2010	1	19	1	5	26.3	13.5
		2014	1	18	0	0	0	
Osaka	Holstein	2016	2	24	2	7	29.2	29.2
Miyazaki	Japanese Black	2013	42	879	26	272	30.9	30.9
Kagoshima	Japanese Black	2016	1	12	1	6	50.0	50.0
Total			≥166	1267	≥83	386	30.5	30.5

For the serological survey, an HI test was conducted using D/swine/Oklahoma/1334/2011. Samples with HI titers ≥40 were defined as positive.

### Serology

Hemagglutination (HA)-inhibition (HI) test was performed to detect the anti-influenza D virus antibody in the bovine serum or plasma samples, according to the World Health Organization manual on animal influenza diagnosis and surveillance [13]. The samples were treated with receptor-destroying enzyme (RDEII; Denka Seiken, Japan) at 37°C for 16 h, followed by heat-inactivation at 56°C for 30 min. Serially diluted samples were then reacted to the D/OK virus (4 HAU) for 30 min at room temperature, followed by incubation with a 0.5% suspension of turkey red blood cells for 30 min at room temperature before reading the result. The HI titer of each sample was expressed as the reciprocal of the highest sample dilution that completely inhibited HA. The samples showing an HI titer ≥40 were considered as positive. An HI titer of 40 has been commonly used as the threshold for a seropositive result in influenza D virus surveillance [3, 8, 9]. This threshold authenticates reaction specificity in HI test [14].

### Statistical Analysis

Using EZR software [15], we statistically compared the positive rates of influenza D virus infection between Holstein and Japanese Black beef cattle by the Chi-squared test, and the numbers of positive samples in each HI titer between these two cattle species by the Mann-Whitney *U* test.

## Results and Discussion

Recently, we reported for the first time, the presence of influenza D virus infection in a dairy cattle herd in Ibaraki Prefecture in central Japan [8]. In this study, we conducted a nationwide serological survey to detect the influenza D virus infection in cattle. To this end, we used the HI test with the reference strain, D/OK. A previous report has shown that there are two HA antigenic groups among influenza D virus strains: the D/OK-type and the D/bovine/Oklahoma/660/2013 (D/660)-type viruses. The former react with antisera against both types of viruses, whereas the latter only react with the homologous D/660-type viruses in HI tests [16]. In our recent report, sera from Ibaraki cows were found to react with both types of viruses and more strongly with D/OK than with D/660-type viruses, suggesting that the influenza D virus found in Japan may possess D/OK-type antigenicity [8]. Based on these data, we selected D/OK as the antigen for our serological survey using the HI test to detect infected animals in this study.

Our assay showed the presence of sero-positive cattle in all the years and in all the prefectural regions tested, with a total positivity rate of 30.5% (386 positives out of 1267 samples), where the positivity rates varied across regions (13.5–50.0%) (Table 1). This strongly suggests that bovine influenza D viruses have circulated for at least 5 years countrywide, emphasizing their ubiquitous distribution in the cattle population of Japan. A serological survey in the USA, found positive samples even among those collected in 2004 [3], suggesting that the influenza D virus is not an emerging virus and that it may be an “old” virus that has been lurking until its first detection in 2011 [1]. Additionally, a metagenomic analysis using the recent developments in gene sequencing technology may help to detect this lurking virus.

**Fig 2:**
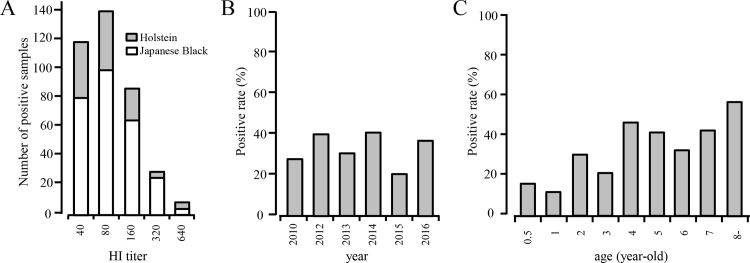
Influenza D virus infection in Japan. (A) The number of positive samples for each HI titer in Holstein (n = 326) and Japanese Black (n = 941) cattle. (B) Positivity rate (%) of cattle samples according to year of collection. (C) Positivity rate (%) according to cattle age.

**Fig 3:**
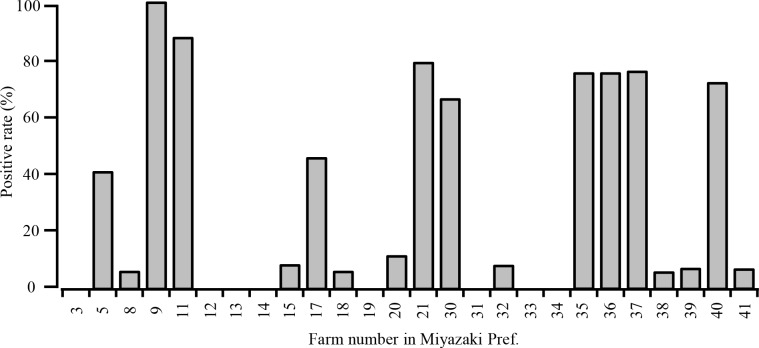
Influenza D virus infection in Miyazaki Prefecture. Positivity rate (%) of cattle in each farm accommodating more than 10 animals is shown.

There was no significant difference in the positivity rates for the Japanese Black (30.6%) and Holstein (30.1%) cattle, and in their ranges of HI titers (Fig 2A). We found that even samples collected in 2010 were antibody-positive (Table 1; Fig 2B), indicating the possible presence of an influenza D virus before 2010 in Japan. When we analyzed sero-positive cattle with age records at the time of collection, positivity rates tended to increase with cattle age, in which nearly half the older cattle were sero-positive (Fig 2D). Interestingly, the herds in Miyazaki were clearly divided into two groups: those with either a high or low (or no) positive rate (Fig 3A and 3B). This was not attributed to the geographic location of the herds in the prefecture (data not shown). We could not determine the reason why two such groups of herds were observed because of the complicated inland movement of cattle. Nonetheless, this finding indicates that horizontal transmission of the virus readily occurs within a herd. Indeed, we have previously encountered an influenza D virus infection, in which the virus spread throughout the herd in a short time [8]. This highly contagious property of the influenza D virus suggests difficulties in controlling its respiratory infection once the virus is introduced into a herd with immunologically naïve cattle.

We have reported that the HEF gene of the Ibaraki strain (D/bovine/Ibaraki/7768/2015) was phylogenetically distant from the D/OK-lineage group, even though this strain possessed D/OK-type HA antigenicity [8]. It is therefore likely that other viruses with HA antigenicities different from D/OK may be circulating in Japan, suggesting that the HI test used in this study may not be able to detect part of the infected cattle. Although the HI test is a reliable method for influenza sero-epidemiology, other serological assays such as ELISA with type-specific NP antigen are required for the broad detection of influenza D virus infection.

The epidemiological data obtained so far and an experimental infection study [17] has indicated that the virus is possibly associated with mild respiratory illness in cattle. However, its pathogenicity appears to be lower than those of other bovine respiratory pathogens such as bovine herpesvirus 1, bovine respiratory syncytial virus, bovine parainfluenza virus 3, bovine viral diarrhea virus, and bovine adenovirus [4, 18]. Currently, several vaccines against these bovine respiratory agents are available in Japan. However, the protective effects of these vaccines are not satisfactory in some cases with respiratory illness, indicating a possibility that these cases may be associated with influenza D virus infection. Further epidemiological studies and virological characterization of influenza D viruses are important to assess its contribution to the bovine respiratory disease complex and to consider vaccine development for this new agent.
